# Evaluation of Serum Vascular Adhesion Protein-1 as a Potential Biomarker in Thyroid Cancer

**DOI:** 10.1155/2016/6312529

**Published:** 2016-06-29

**Authors:** Zhigang Hu, Pengxin Zhao, Kaili Zhang, Leilei Zang, Haiying Liao, Weiyuan Ma

**Affiliations:** The Second Hospital of Hebei Medical University, No. 215 Heping West Road, Shijiazhuang, Hebei 050000, China

## Abstract

Vascular adhesion protein-1 (VAP-1) is a glycoprotein that mediates tissue-selective lymphocyte adhesion. The prognostic value of VAP-1 has been determined in gastric cancer. The aim of this study was to evaluate the changes and the predictive value of serum VAP-1 in patients with thyroid cancer. A total of 126 patients with thyroid nodules and 53 healthy controls participated in this study. The patients were further divided into subgroup 1 (69 cases with benign thyroid nodules) and subgroup 2 (57 cases with thyroid cancer). Serum VAP-1 was measured by time-resolved immunofluorometric assay. Diagnostic value of presurgical VAP-1 for thyroid cancer was conducted by receiver operating characteristic (ROC) curves. Serum levels of VAP-1 were significantly lower in thyroid cancer group than in healthy control and benign thyroid nodule groups. VAP-1 concentrations negatively correlated with serum thyroglobulin (Tg) levels in thyroid cancer patients (*r* = −0.81; *p* < 0.001). The optimum cut-off value of VAP-1 was 456.6 ng/mL with a 77.4% specificity and 66.7% sensitivity for thyroid cancer diagnosis. Serum VAP-1 decreased in thyroid cancer patients and VAP-1 could be a potential useful adjunct biomarker in the diagnosis of thyroid cancer.

## 1. Introduction

Thyroid cancer is one of the most common head and neck cancers with increasing incidences all over the world [1]. The five-year survival rate across all the stages is 97%, but they are different among the stages. The five-year survival is only 59% in a late stage compared to nearly 100% for an earlier, localized stage [2]. Early detection is one of the keys to reduce the mortality. At present, fine-needle aspiration cytology (FNAC) is the most commonly used diagnostic tool for diagnosing thyroid diseases, especially for small nodules (<1.5 cm) and nonpalpable thyroid nodules [3]. Although FNAC is a primary method for detecting malignant nodules, 10%–25% of thyroid nodules are categorized as indeterminate nodules [4]. Studies have shown that serum thyroglobulin (Tg) determination serves as the proper monitoring strategy for differentiated thyroid cancer (DTC) after surgery [5, 6]. However, some studies demonstrated undetectable preoperative Tg in patients with DTC [7] and low postoperative nonstimulated Tg in patients with ^131^I-avid metastases [8]. Hence, it is necessary to screen sensitive presurgical biomarkers for predicting thyroid cancer.

Vascular adhesion protein-1 (VAP-1), a 170-kDa homodimeric glycoprotein, is an endothelial adhesion molecule involved in leukocyte rolling, adhesion, and transmigration into sites of inflammation [9]. Another function of VAP-1 is as an enzyme, semicarbazide-sensitive amine oxidase, which catalyzes oxidative deamination of primary amines into aldehydes, hydrogen peroxide, and ammonia [10, 11]. VAP-1 has a soluble circulating form, which retains its enzymatic function. Serum VAP-1 is released or shed from many tissues such as endothelium, adipocyte, and smooth muscle cells [12, 13]. VAP-1 is upregulated at sites of inflammation, and it mediates lymphocyte binding to inflamed endothelium [14, 15]. Increased serum VAP-1 levels in chronic liver disease and multiple sclerosis compared to healthy individuals have been reported previously [16, 17]. In addition, low serum VAP-1 is associated with disease relapse and worse prognosis in colon cancer and gastric cancer [18–21]. However, the diagnostic and prognostic values of serum VAP-1 for TC, particularly before surgery, have not yet been studied.

In this study, we determined the serum levels of VAP-1 in the thyroid cancer and benign thyroid adenoma patients. We further evaluated the prognostic value of serum VAP-1 in patients with thyroid cancer.

## 2. Materials and Methods

### 2.1. Study Population

In this study, 179 participants were recruited from May 2013 to July 2015 in The Second Hospital of Hebei Medical University, including 53 healthy controls (volunteer), 69 patients with benign thyroid nodules, and 57 patients with thyroid cancer. The 53 healthy participants have no diseases reported, who had come to receive their annual health checkup. Clinical characteristics and demographic data were collected by retrospective chart review. Patients with the following conditions were excluded: (a) autoimmune disease; (b) coexistence of other cancers in addition to thyroid cancer; (c) pregnancy; (d) positive thyroglobulin antibody, because the serum thyroglobulin concentration is often subject to interference of thyroglobulin antibodies, leading to false-negative occurrence [1]. Physical exam, biopsy, and imaging tests (ultrasound, CT scan, MRI, chest X-ray, and nuclear medicine scans) were performed in each patient before surgery. In the surgical cohort, 126 cases with thyroid nodules were recruited. 69 patients were diagnosed with benign thyroid nodules (42 cases of multinodular goiter and 27 cases of thyroid adenoma) as the thyroid was normal in tissue biopsy and thyroid ultrasonography tests. 57 patients were diagnosed with thyroid cancer as positive cytological results for samples obtained by preoperative fine-needle aspiration from enlarged cervical neck lymph nodes. The pathologic classification of 57 patients with thyroid cancer was based on the original surgical pathology report, including 46 cases of papillary histotype (PTC) and 11 cases of follicular thyroid carcinoma (FTC). The TNM classification system of the American Joint Committee on Cancer (AJCC) was used for staging [22]. Serum VAP-1 and other parameters were also tested before operation. Participants with benign thyroid nodules received either lobectomy or nodule resection. All the participants with thyroid cancer received total thyroidectomy and neck lymph node resection if metastasis was suspected. The study was approved by the Ethics Committee of The Second Hospital of Hebei Medical University and informed written consent was obtained from all study participants.

### 2.2. Collection of Serum

Peripheral venous blood samples were collected from all participants in fasting state and were gently subjected to centrifugation at 1500 ×g for 15 min. Serum supernatants were collected, aliquoted, and stored at −80°C until use. None of the patients received any preoperative radiotherapy, chemotherapy, or blood transfusions. All of the samples were taken in accordance with the regulations and approval of the institutional review board of The Second Hospital of Hebei Medical University.

### 2.3. Laboratory Measurements

All experiments were performed in accordance with relevant guidelines and regulations. Thyroglobulin (Tg) was assessed by a chemiluminescent reaction on a fully automated IMMULITE 2000 analyzer (Siemens Healthcare Diagnostics, Los Angeles, USA). Free thyroxine (FT4) and thyroid stimulating hormone (TSH) assays were performed on a fully automated ADVIA Centaur analyzer (Siemens Healthcare Diagnostics, New York, USA). These assays were based on the chemiluminescent reaction principle.

### 2.4. Measurement of Serum VAP-1

Serum VAP-1 was measured by time-resolved immunofluorometric assay as stated previously [23]. Briefly, the assay utilized a biotin-conjugated monoclonal anti-human VAP-1 antibody (Biotie Therapies Corp.) as a capturer on a streptavidin-coated microtiter plate. Detection of bound serum VAP-1 was performed using a different europium-conjugated anti-human VAP-1 antibody (Biotie Therapies). The time-resolved fluorescence was measured using a fluorometer (Victor2 Multilabel Counter, PerkinElmer) at 615 nm. Serum VAP-1 concentration was quantified on the basis of a reference sample of highly purified human serum VAP-1 (Biovian Ltd.). The intra- and interassay coefficients of variation were <5% and <10%, respectively. All samples were measured in triplicate.

### 2.5. Statistical Analysis

Data are presented as mean ± SD unless otherwise indicated. The data was analyzed by a two-tailed unpaired *t*-test. Correlation analysis was performed using nonparametric Spearman's correlation test. Serum VAP-1 concentrations were used to draw receiver operating characteristic (ROC) curve, and the specificity, sensitivity, and area under the ROC curve (AUC) and cut-off value were determined. The AUCs were used to estimate model performance. A *p* value < 0.05 was considered statistically significant. All statistical analyses were performed using Prism (GraphPad Software, Inc.).

## 3. Results

### 3.1. Characteristics of the Study Subjects

A total of 126 patients with thyroid nodules and 53 healthy controls were enrolled in this study. The patients were further divided into subgroup 1 (69 cases with benign thyroid nodules) and subgroup 2 (57 cases with thyroid cancer). Among the thyroid cancer patients, 41 (71.9%) had tumor stage I/II and 16 (28.1%) had tumor stage III/IV. The baseline characteristics of participants are summarized in Table 1. The age and gender distribution for healthy controls, benign thyroid adenoma, and thyroid cancer patients were not statistically different. Serum Tg levels were found to be significantly higher in the thyroid cancer patients than in the normal control subjects. No significant difference was found in other biochemical results, including FT4 and THS between patient and control group. In addition, no significant difference was observed in diabetes and hepatic diseases between patients with benign thyroid nodule and with thyroid cancer.

### 3.2. Decreased Serum Level of VAP-1 in Thyroid Cancer Patients

**Figure 1:**
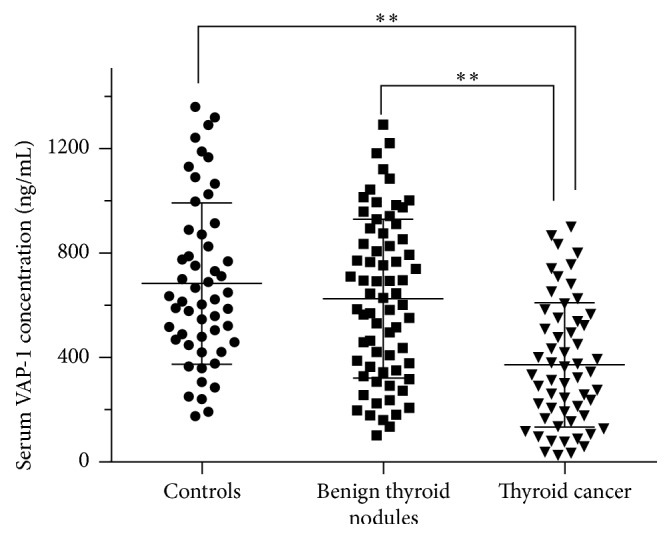
Serum level of VAP-1 in patients and healthy controls. VAP-1 concentrations were measured in serum from healthy controls (*n* = 53), patients with benign thyroid nodules (*n* = 69), and patients with thyroid cancer (*n* = 57). Scatter graphs were plotted to demonstrate the intergroup differences of presurgical serum VAP-1 in patients as well as in control subjects. Data are presented as mean ± SD. ^*∗∗*^ *p* < 0.01.

To determine the changes of VAP-1 in patients with thyroid cancer, the serum levels of VAP-1 were measured in healthy controls, patients with benign thyroid nodules, and patients with thyroid cancer. Scatter graphs of VAP-1 were plotted to demonstrate the intergroup differences (Figure 1). The serum VAP-1 levels were significantly lower in thyroid cancer patients than in healthy controls, as well as that in the benign thyroid nodule patients. These data suggest that VAP-1 is involved in the pathogenesis of thyroid cancer.

### 3.3. VAP-1 Concentration Is Associated with Tg Level

**Figure 2:**
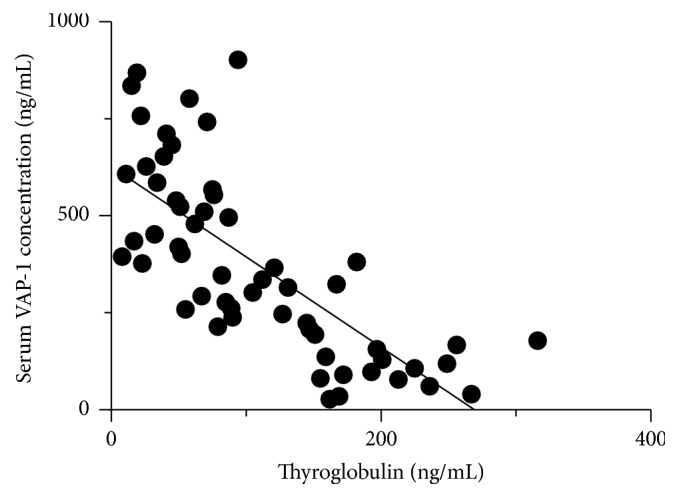
The correlation between serum VAP-1 concentration and Tg level. The correlations between serum VAP-1 concentration and thyroglobulin (Tg) level in thyroid cancer patients were examined using Spearman's correlation coefficient.

Since preoperative increasing serum levels of Tg correlate with higher risk of thyroid cancer [24, 25], we analyzed the correlation between the serum VAP-1 concentrations and Tg levels in thyroid cancer patients. As shown in Figure 2, nonparametric Spearman's correlation test showed that a negative correlation was observed between the serum VAP-1 concentration and levels of Tg (*r* = −0.81; *p* < 0.001). We further analyzed the relationship of serum VAP-1 concentrations with age and tumor stage using Spearman's correlation test. No significant correlation was found between VAP-1 concentrations and age (*p* = 0.62) and tumor stage (*p* = 0.12), respectively.

### 3.4. ROC Analysis of VAP-1 Diagnostic Value for Thyroid Cancer

**Figure 3:**
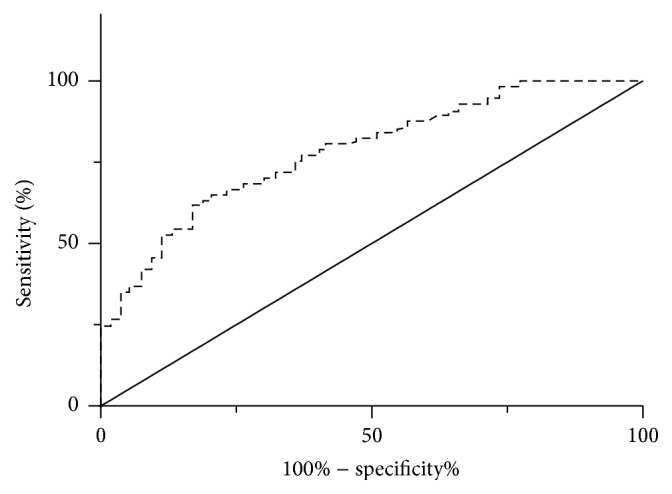
ROC curve. Serum VAP-1 levels in thyroid cancer and healthy controls were used to draw ROC curve, and the specificity, sensitivity, area under the ROC curve (AUC), and cut-off value were determined.

To evaluate the predictive value of VAP-1 for thyroid cancer, we performed ROC curve analysis. As shown in Figure 3, the area under the ROC curve was 0.78 (95% confidence interval, 0.70–0.87; *p* < 0.001). The decision on optimal cut-off value for serum VAP-1 was based on maximizing the sum of sensitivity and specificity. The cut-off value of VAP-1 was 456.1 ng/mL with a 77.4% specificity and 66.7% sensitivity for thyroid cancer diagnosis. These data suggest that serum VAP-1 has a good capability in predicting thyroid cancer.

## 4. Discussion

It has been over 40 years since biological markers were first introduced as a way to detect and manage thyroid cancer [26]. From the beginning, the goal was to find markers that can identify benign thyroid nodules and malignant thyroid tumors and to predict the behavior of these thyroid cancers. However, thyroid nodules remain a challenge from both a diagnostic and a management perspective [27]. An accurate presurgical biomarker that can quantify the likelihood of malignancy within a thyroid nodule is needed. In this study, we found that serum VAP-1 levels were significantly lower in thyroid cancer group than in healthy control and benign thyroid nodule groups. Serum VAP-1 level negatively associated with serum thyroglobulin concentration in thyroid cancer patients and has a good capability in the diagnosis of thyroid cancer. These findings suggest that serum VAP-1 could be a potential useful adjunct in the diagnosis of thyroid cancer.

VAP-1 is one of the endothelial adhesion molecules which supports shear-dependent lymphocyte binding to high-endothelial venules in lymph nodes [14, 28]. A number of studies have suggested a strong correlation between serum VAP-1 levels and cancer prognosis [18, 19, 21]. One recent study showed that the serum VAP-1 level was significantly lower in the colorectal cancer group compared with the control group. Tissue VAP-1 protein and mRNA levels were also significantly lower in colorectal cancer compared with normal colon tissue. VAP-1 immunostaining was practically absent from colorectal cancer. These findings suggest that VAP-1 is downregulated in human colorectal cancer and it may be part of a mechanism used by the tumor to prevent the recruitment of antitumor immune cells [20]. In addition, Li et al. reported that serum VAP-1 predicts mortality independently and improves risk stratification in colorectal cancer subjects [29]. In contrast, strong VAP-1 expression has been detected on liver cancers where it has been proposed that VAP-1 supports the recruitment of lymphocytes [30]. Increased VAP-1 immunoreactivity has been demonstrated in microvessels in human conjunctival tumors [31] and in human breast cancer, tumor VAP-1 mRNA expression is associated with oestrogen receptor expression and improved prognosis [32, 33]. Interestingly, Yasuda et al. demonstrated that serum VAP-1 levels are significantly higher in gastric cancer patients compared to healthy controls. Furthermore, an inverse correlation between serum VAP-1 level and tumor size, serosal invasion, lymph node metastasis, peritoneal dissemination, and TNM classification was found, and patients with low VAP-1 levels had a significantly poorer prognosis compared to those with high VAP-1 levels [21]. There is limited data about the clinical importance of serum VAP-1 levels in thyroid cancer. To the best of our knowledge, this is the first report which analyzes serum VAP-1 levels in thyroid cancer patients. Decreased serum VAP-1 concentration was found in patients with thyroid cancer group compared to healthy control and benign thyroid nodule groups.

Serum biomarkers represent the first generation of thyroid biomarkers. Ideally, a serum biomarker is one that is highly sensitive and specific, can establish diagnostic certainty, and can be easily measured [34]. This definition has remained fairly consistent over several decades, despite the introduction of complex bioinformatics systems that are being used as analytical tools to identify new biomarkers. In recent years, several immunohistochemical markers, such as cytokeratin-19, galectin-3, HBME-1, fibronectin-1, and intracellular sodium/iodide symporter, or their combinations have been used to differentiate benign thyroid diseases from thyroid cancer [35–37]. Another study has indicated that diffusion-weighted MR imaging as a promising noninvasive method to diagnose diseases has high diagnostic ability to differentiate benign from malignant in thyroid lesions [38, 39]. However, diffusion-weighted MR imaging technology is very complex and easily affected by instrument parameters. In the present study, a negative correlation between the serum VAP-1 and thyroglobulin levels was observed. Previous studies have indicated that preoperative serum thyroglobulin levels could be a predictor of differentiated thyroid cancer [24, 25, 40]. Al-Bader et al. also reported that thyroglobulin levels can aid in preoperative assessment of a thyroid nodule [41]. However, Gupta et al. showed that high serum thyroglobulin with negative ^131^I whole body scan did not warrant an aggressive differentiated thyroid cancer disease. These patients in their 5 years' or till recurrence follow-up showed downward trends of serum thyroglobulin, no higher risk or recurrence, and no shorter progression-free survival [42]. Moreover, the role of VAP-1 in tumor progression is likely to depend on both the host and tumor type [21]. Thus, the relationship between serum VAP-1 and thyroid cancer is independent of thyroglobulin.

Another important finding of our study is that the optimum cut-off value of VAP-1 was 456.6 ng/mL with a 77.4% specificity and 66.7% sensitivity for thyroid cancer diagnosis, suggesting that serum VAP-1 had relatively high sensitivity and specificity in predicting thyroid cancer. A number of recent studies have showed that serum biomarkers play important role in the diagnosis of thyroid cancer. For instance, Zheng et al. reported that higher serum thyroid stimulating hormone concentration is associated with an increased risk of thyroid cancer [43]. Hedayati et al. showed that myostatin serum levels may have a potential ability for early diagnosis of cachexia in medullary thyroid cancer patients, especially in females [44]. Cho et al. demonstrated that one-third of the patients with medullary thyroid cancer with hypercalcitoninemia experienced structural recurrence, and postoperative basal serum calcitonin might be a simple tumor marker to predict structural recurrence [45]. In addition, it has been reported that low Wnt inhibitor Dickkopf-1 serum levels are associated with poor prognosis in papillary thyroid cancer patients and Dickkopf-1 could potentially be used as a biomarker leading to earlier diagnosis of papillary thyroid cancer [46]. Interestingly, one recent study showed that the combined application of US-guided fine-needle aspiration cytology and thyroglobulin measurement on fine-needle aspiration/serum thyroglobulin contributes to improving the accuracy of diagnosing cervical lymph node metastases in patients with thyroid cancer [47]. Our study showed that serum VAP-1 could be a potential useful adjunct in the diagnosis of thyroid cancer. Therefore, the combined application of ultrasonographic features, reports of aspiration cytology or biopsy, and VAP-1 measurement could be a potential way to improve the accuracy of diagnosing thyroid cancer.

There are several limitations in this study, which warrant further research. First, we recruited a relatively small number of cases and conducted a retrospective study. Second, we did not perform follow-up analyses on prognostic values of VAP-1 which predict new metastases or recurrence. Third, the patients with positive thyroglobulin antibody were excluded and this study included only Chinese subjects; therefore, our results may not fully apply to the general patient. Thus, large prospective longitudinal studies, ideally involving many ethnic groups, are needed to improve our understanding of the long-term health risks associated with thyroid cancer.

In conclusion, decreased preoperative serum VAP-1 levels were found in patients with thyroid cancer. Serum VAP-1 could be a useful adjunct in the diagnosis of thyroid cancer. However, these data are exploratory and preliminary. Future larger prospective cohort studies, which include postoperative levels of VAP-1 and longer follow-up periods, will better define the clinical utility and prognostic use of this marker.

## Figures and Tables

**Table 1:** Characteristics of participants and their laboratory values.

Characteristics	Healthy controls (*n* = 53)	Patients with benign thyroid nodule (*n* = 69)	Patients with thyroid cancer (*n* = 57)
Age (years)	47.7 ± 16.2	48.6 ± 18.4	49.5 ± 20.1
Male (*n*, %)	25 (47.2%)	34 (49.3%)	31 (54.4%)
Female (*n*, %)	28 ( 52.8%)	35 (50.7%)	26 (45.6%)
Tg (ng/mL)	10.3 ± 11.4	16.4 ± 14.3	109.3 ± 76.3^*∗∗*^
FT4 (pmol/L)	14.2 ± 4.1	15.1 ± 3.9	16.5 ± 4.6
TSH (*μ*IU/mL)	3.4 ± 1.9	3.5 ± 2.1	3.7 ± 3.2
VAP-1 (ng/mL)	683.7 ± 309.2	625.7 ± 304.1	372.3 ± 238.5^*∗∗*^
Diabetes (*n*, %)	—	4 (5.8%)	5 (7.2%)
Hepatic disease (*n*, %)	—	3 (4.3%)	2 (3.0%)
Thyroid cancer stage			
I/II (*n*, %)	—	—	41 (71.9%)
III/IV (*n*, %)	—	—	16 (28.1%)

Data are presented as the mean ± SD. ^*∗∗*^ *p* < 0.01 versus control. Tg: thyroglobulin; FT4: free thyroxine; TSH: thyroid stimulating hormone. VAP-1: vascular adhesion protein-1.
